# The Predictability of Phytophagous Insect Communities: Host Specialists as Habitat Specialists

**DOI:** 10.1371/journal.pone.0025986

**Published:** 2011-10-07

**Authors:** Jörg Müller, Jutta Stadler, Andrea Jarzabek-Müller, Hermann Hacker, Cajo ter Braak, Roland Brandl

**Affiliations:** 1 Bavarian Forest National Park, Grafenau, Germany; 2 Chair for Terrestrial Ecology, Research Department Ecology and Ecosystem Management, Technische Universität München, Freising-Weihenstephan, Germany; 3 Department of Community Ecology, Helmholtz Centre for Environmental Research, UFZ Leipzig-Halle Ltd., Halle, Germany; 4 Bavarian State Collection of Zoology, Munich, Germany; 5 Biometris, WUR, Wageningen, The Netherlands; 6 Animal Ecology, Department of Ecology, Faculty of Biology, Philipps University Marburg, Marburg, Germany; French National Centre for Scientific Research, Université Paris-Sud, France

## Abstract

The difficulties specialized phytophagous insects face in finding habitats with an appropriate host should constrain their dispersal. Within the concept of metacommunities, this leads to the prediction that host-plant specialists should sort into local assemblages according to the local environmental conditions, i.e. habitat conditions, whereas assemblages of host-plant generalists should depend also on regional processes. Our study aimed at ranking the importance of local environmental factors and species composition of the vegetation for predicting the species composition of phytophagous moth assemblages with either a narrow or a broad host range. Our database consists of 351,506 specimens representing 820 species of nocturnal Macrolepidoptera sampled between 1980 and 2006 using light traps in 96 strict forest reserves in southern Germany. Species were grouped as specialists or generalists according to the food plants of the larvae; specialists use host plants belonging to one genus. We used predictive canonical correspondence and co-correspondence analyses to rank the importance of local environmental factors, the species composition of the vegetation and the role of host plants for predicting the species composition of host-plant specialists and generalists. The cross-validatory fit for predicting the species composition of phytophagous moths was higher for host-plant specialists than for host-plant generalists using environmental factors as well as the composition of the vegetation. As expected for host-plant specialists, the species composition of the vegetation was a better predictor of the composition of these assemblages than the environmental variables. But surprisingly, this difference for specialized insects was not due to the occurrence of their host plants. Overall, our study supports the idea that owing to evolutionary constraints in finding a host, host-plant specialists and host-plant generalists follow two different models of metacommunities: the species-sorting and the mass-effect model.

## Introduction

Four factors constrain the composition of local assemblages, i.e. the co-occurrence of species of a taxon [1]: the regional species pool, the connectivity of the habitat, local environmental filters and local biotic interactions [2], [3], [4], [5]. Firstly, local assemblages are embedded in a regional setting, and the regional species pool sets the framework for the composition of local assemblages [6], [7], [8]. Secondly, species of the pool are only able to arrive at a particular habitat if the considered habitat is sufficiently connected to other habitats occupied by the species [9]. Of course, the connectivity of a habitat varies from species to species, depending on the mobility, dispersal propensity and dispersal strategy. Dispersal to and from a habitat also modifies the local abundance of species [9], [10]. Thirdly, local environmental conditions act as filters, and only arriving species able to cope with the local conditions can pass these filters [11]. Fourthly, local biotic interactions (e.g. competition, predation, herbivory or mutualisms) determine the occurrence and modify the abundance of species [12]. The ranking in importance of these factors for the composition of assemblages in a habitat is still a matter of debate [13], [14], [15].

Assemblages of phytophages are only rarely structured by competitive interactions [16]. Therefore, given a regional set of species and a set of habitats, the environmental filters [17], [18], the host relationships and dispersal should set important constraints for the composition of local assemblages [16], [19]. Furthermore, the dispersal of the species traits and host use may not be independent. A considerable number of theoretical studies of dispersal have suggested that dispersal is selected for by a temporal variability of habitat quality, e.g. [20], [21]. Furthermore, spreading of risk in stochastic environments leads to a joint evolution of low dispersal and habitat specialization [22]. Dispersing phytophages have the problem of locating patches with appropriate host plants [23], and the importance of this predicament may differ between host-plant generalists and specialists. For specialized phytophages, it may be more dangerous to leave a patch with suitable host plants than for generalists. Furthermore, for insect species with adaptive host-plant selection, the adult lifespan of females should be negatively correlated with the number of host plants used by the larvae; indeed, this has been found for many species of Lepidoptera [24]. This suggests that specialization constrains search time [25]. Overall, in host-plant specialists there should be either a selection against high mobility [26], [27] or effective search strategies for finding habitats with suitable hosts. Therefore specialists may also evolve to habitat specialists. In contrast, for host-plant generalists, suitable hosts occur almost everywhere, and finding habitat patches suitable for reproduction is less dangerous than for specialists. Overall they may become habitat generalists. The higher mobility and dispersal of adult generalists (for butterflies, see [26], [28], [29]) allows exchange between habitat patches with appropriate abiotic conditions, and as a consequence, the dynamics of generalists in a patch depends not only on processes within this patch but also on local processes in neighbouring patches and the connectivity between patches [30]. These arguments suggest that the dispersal strategy and thus the linkage between patches with its implications on the ecological processes within local assemblages depend — owing to a trade-off between dispersal and specialization — on diet specialization [26], [31], [32].

The metacommunity concept [10], [33] is a powerful tool to understand assemblages in their regional setting, particularly along environmental gradients [33], [34]. Leibold et al. [10] introduced four simplified paradigms of metacommunities (the neutral, the patch-dynamics, the species-sorting and the mass-effect paradigms). The species-sorting paradigm assumes that patches are heterogeneous in some environmental factors. Species assemble in local communities according to the local environmental factors and niche characteristics, and the strength of dispersal is insufficient to alter distributions. In contrast, the mass-effect paradigm implies that owing to dispersal, species are present in source and sink habitats, and the composition of local assemblages is more or less independent of local environmental factors [10]. These two models are of course extremes. However, generalists should fit more to the mass-effect paradigm, whereas specialists should fit more to the species-sorting paradigm. Concentrating on the issue of the dispersal strategies of specialists and generalists, we hypothesize that local assemblages of specialists should be easier to predict from local environmental factors than local assemblages of generalists.

Local factors can be measured by environmental variables but also by the species composition of the vegetation [35]. The vegetation may even be a far better predictor of insect assemblages than environmental factors or measures of vegetation structure [35]. Clearly, for associations of specialized insects and host plants, the species composition of the vegetation sets a frame for the occurrence of these insects, and we expect that besides habitat conditions also host-plant relationships drive the relationship between the species composition of assemblages of specialized insects and of plants [35], [36], [37], [38]. However, we expect the host-plant generalists and specialists to differ in the ranking of the importance of environmental factors versus host relationships for the composition of whole assemblages of phytophages. Two statistical methods for such an analysis — predictive co-correspondence analysis and predictive canonical correspondence analysis — have been developed by Ter Braak & Schaffers [39]. We used these two methods to test the following hypotheses concerning the predictability of local species assemblages of specialists and generalists:

1. We expect that the predictability of assemblages of phytophagous insects by local environmental factors is higher for host-plant specialists than for host-plant generalists.
2. For host-plant specialists, the predictability of assemblages of phytophagous insects from the assemblage of host plants exceeds the predictability from environmental factors.

## Materials and Methods

### Sampling of moths

**Figure 1:**
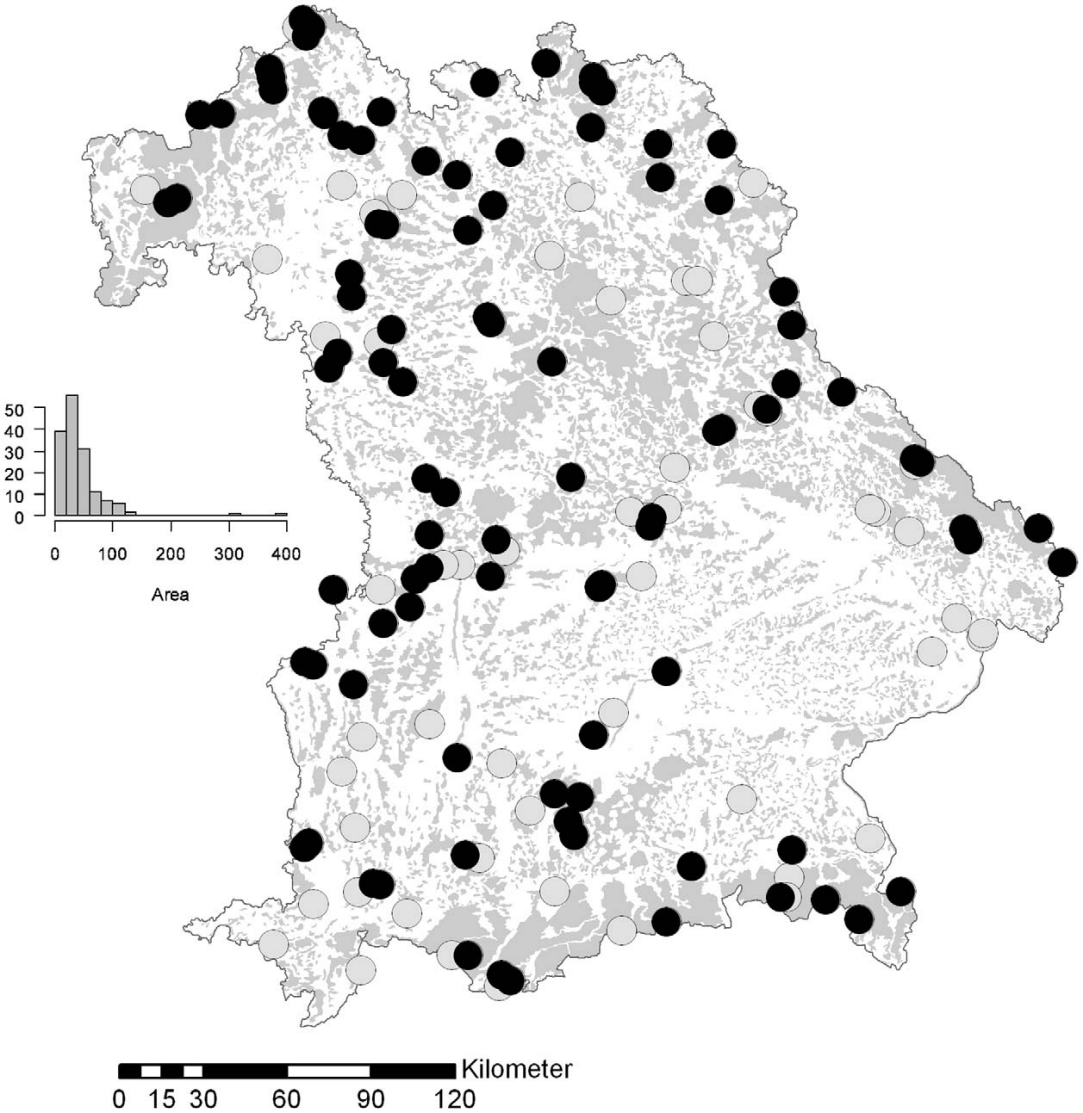
Distribution of the 154 strict forest reserves in Bavaria. The histogram shows the distribution of the area covered by each reserve (ha). The 96 reserves included in the analyses are shown as black dots; the reserves not included in the analyses are shown as grey dots. Grey shading indicates forested area. Lines indicate borders between the forest ecoregions used in Fig. S3.

Since 1978, authorities in Bavaria, Germany, have stopped logging in 154 remnants of natural forests, and programs have been launched to monitor assemblages of organisms in these forests (e.g. [40]). Insects were collected with light traps in 114 of these strict forest reserves in Bavaria between 1980 and 2006 (Fig. 1; see [40]). All nocturnal Macrolepidoptera were identified to the species level, and are, referred to as moths for simplicity (for raw data see Tab. S2, S3, S4).

Some reserves were sampled only during one night, and other reserves were sampled up to 38 times over up to 8 years (Table S1). Although the sampling effort among the reserves varied considerably, we decided for the present analyses to pool all trap nights for each site for a reliable estimate of the relative abundance of species. Differences in sampling effort are common in studies of invertebrates on larger scales, and pose problems for the analyses [41]. A plot of species richness versus sampled individuals showed a curvilinear relationship with a decrease in the slope at around 500 individuals (Fig. S1a). Using two methods to extrapolate the total number of species, we found that for reserves where fewer than 500 individuals were sampled, the ratio of sampled to expected species varied considerably (Fig. S1c). Therefore, for the present analyses, we selected reserves with a minimum of 500 sampled individuals. Furthermore, when we used the number of trapping nights to check for insufficiently sampled reserves, we found that our decision to use only reserves with at least 500 sampled individuals also removed sites with few trapping nights (Fig. S1e, f). This selection boiled our primary data set down to 96 reserves with 820 species (Fig. 1). The mean percentage of unsampled species in these reserves was only 18% (range 1–33%; Fig. S1c,f).

It has also been repeatedly shown that abundances of moths can fluctuate considerably with time [42]. However, the influence of such variations on measures of diversity for our data set was low. We divided the total time span into periods of five years (see Fig. S2), and we estimated the additive between-period component of β-diversity for various measures of diversity (Fig. S2). We are aware of the discussions associated with additive partitioning of diversity [43], [44], [45]. Nevertheless, this analysis showed clearly that the between-period component of β-diversity was much lower than other components (for details, see Fig. S2).

**Figure 2:**
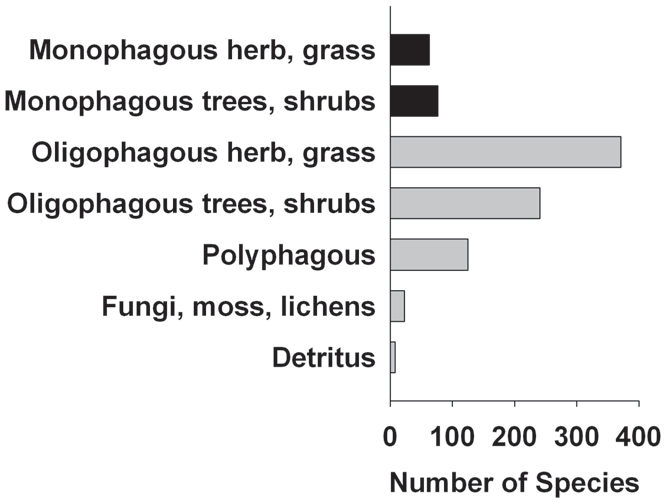
Number of species of nocturnal Macrolepidoptera occurring in Bavaria with different levels of specialization with respect to the host plant of the larvae (monophagous: 1 plant genus; oligophagous: 2–3 plant genera; polyphagous: more than 3 plant genera). Black bars indicate groups classified as specialists in the present study.

We grouped the species into two categories — specialists and generalists — using the compilation of host plants in Central Europe (see [40]; Table S3). We considered species as specialists when their larvae feed on species of one plant genus (Fig. 2). All other species, including species feeding on fungi, bryophytes, lichens (all of which feed on several genera) and detritus, were classified as generalists. Furthermore, we restricted our analyses to moth species occurring in more than five reserves (see Fig. 2), which resulted in 571 moth species, 79 of which were specialists (see Table S2 for raw data).

### Environmental predictors

1. We arranged the variables used to predict the composition of assemblages into two sets: environmental data and the composition of the vegetation (see Tables S3 and S4 for raw data). The environmental data set comprised 18 variables characterizing climate and soil conditions. Variables characterizing the climate were scores of a correlation-based principal component analysis of 19 bioclimatic variables available in an open source atlas [46]. We used the first three axes for further analyses, accounting for 89% of the total variability. Additionally, we used the mean altitude of each reserve [47] to characterize the macroclimatic conditions. From vegetation relevés (see below), we calculated mean Ellenberg indicator values for light (L), temperature (T), moisture (F), soil reaction (R), nitrogen (N), and continental climate (K) [48]. These values indirectly characterize both soil and microclimatic conditions [48]. Furthermore, we included a second-order trend surface to consider geographic space using the Gauss-Krüger coordinates.
2. Vegetation data were extracted from the unpublished database of the Bavarian State Institute for Forestry. We considered vegetation data collected only within the same time frame as the insect data. In this database, cover abundance of species is recorded on a modified Braun-Blanquet scale, with ‘+’ coding for <1% cover scale. For further analyses, we recoded this rank scale as follows: r recoded to 0.05%, + to 0.5%, 1a to 2%, 1 and 2 m to 3%, 1b to 4%, 2a to 10%, 2 to 15%, 2b to 20%, 3a to 31%, 3 to 38%, 3b to 44%, 4 to 63%, and 5 to 83%. The sampling effort for plants differed among reserves and ranged from 1 to 137 relevés (mean = 10). We found no correlation of species richness with the number of relevés (see Fig. S3). Therefore, we used all available information and we constructed for each sampled reserve a matrix of all plant species recorded during the relevés. For the final analysis, we scored the presence and absence of plant species that occurred in at least 5 reserves (cf. [35]).

### Predicting the composition of moth assemblages

To predict the composition of the assemblages of generalist and specialist moths or of any other subdivision of our data set, we used a predictive version of direct gradient analysis and predictive co-correspondence analysis [35], [39]. To check for the influence of common species on our analysis, we compared the predictive power of our models using the raw data with results of three different types of transformations or standardizations: log(x+1) transformation, square-root transformation, and a standardization based on the total number of individuals sampled on each site (relative abundance). When relative abundance is analysed with a co-correspondence analysis, all sites have equal weight. In contrast, for the raw data, sites with lower abundances have lower weight. The square-root and log-transformed data give more emphasis to the less frequent moths than the untransformed data set.

We tested the significance of axes and terms using functions available in the packages *vegan* for canonical correspondence analysis and *cocorresp* for co-correspondence analysis, both available in R. As a yardstick of the predictive power of the different analyses, we used “leave-one-out” cross-validation (see [39]) because there were many more predictor plant species than sites and the response data (moth species across sites) can be fitted without error by taking as many axes as sites. Thereby, the number of relevant axes is the number of axes that minimizes the squared prediction error. We followed the method of [39] and reported the cross-validatory fit as 100 (1 − ssp_n_/spp_0_) for n = 1 to 25 axes, where ssp_a_ is the sum of squared prediction error using n axes, and spp_0_ is the sum of the squared prediction errors if rows and columns of the response matrix are independent. The maximum number of axes evaluated was set arbitrarily to 25; this had no influence on our conclusions. The cross-validatory fit may even become negative, which indicates that the prediction using the mean abundance of species is already better than the predictive co-correspondence analysis or canonical correspondence analysis models. The significance of differences in the cross-validatory fit of two different sets of predictors on a response set was tested by a randomization test following van der Voet [49]. The data matrices of specialist and generalist differed considerably in dimension (79 and 492 taxa, respectively), which additionally hampered the comparison between specialists and generalists. Therefore, we randomly selected 79 species from the list of generalists and calculated the cross-validatory fit for n = 1 to 25 axes (100 random draws).

To test our third hypothesis — the influence of host plants on the predictability of specialists — we used two approaches. Firstly, we calculated a number of co-correspondence analyses, one for each moth. For moth *k*, we deleted its host plants from the data and re-computed the cross-validatory fit. This analysis then does not include the host-plant associations of species *k*. We then averaged the fit across the 79 species of specialists. If the moth/host plant association drives the results of the co-correspondence analysis, we would expect a considerable decrease in the cross-validatory fit. Secondly, we compared the explained variance of the regression of a moth species on its host plants with regressions of randomly drawn plant species. The conceptual difficulty is that co-correspondence analysis treats the data as compositional; therefore, we need to do a regression of percentages in which one moth percentage is against all others, and one host percentage is against all others. This kind of regression can be done with co-correspondence analyses with two species in both the response and predictor matrix (response matrix: moth − all other moths; response matrix: host plant(s) − all other plants). We first calculated the mean explained variance across all specialists for the real data and then compared this value to the mean with randomly drawn plant species. For this, for each moth species, we randomly selected from the plant matrix a plant species or several plant species, depending on the original number of host species occurring in our sites, and calculated the mean as for the original data. This procedure was repeated 100 times to generate a distribution of the explained variance, ignoring host–moth associations.

## Results

Across the 96 reserves (Fig. 1), we sampled 351,506 specimens representing 820 species of moths, from which 571 occurred in at least 5 sites. The samples were dominated by oligophagous species with larvae feeding on herbs, followed by oligophagous species with larvae feeding on trees and shrubs (Fig. 2). Of the 820 species, we classified 691 as generalists and 129 as specialists (Fig. 2; 492 generalists and 79 specialists occurred in at least 5 reserves). As expected, mean abundance (the sum of all sampled individuals) of generalists occurring on at least 5 reserves was higher than the mean abundance across specialists (geometric mean generalists = 6058; mean specialists = 3517). Nevertheless, the overlap between specialists and generalists was large (Fig. S1b), and an Anova of log-transformed data indicated only marginal significance (p = 0.06; note that this test ignores phylogenetic relatedness and is therefore only approximate). The distribution which was measured as the number of reserves in which a species was recorded, increased with abundance (Fig. S1b). After correcting for abundance we found no difference in the occupancy between generalists and specialists (p>0.5).

**Figure 3:**
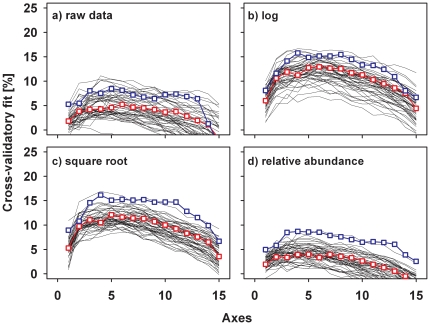
Cross-validatory fit of the raw data set of the composition of moth species and of three types of transformed data using local environmental factors as the predictor. The fit is plotted against the number of ordination axes. The blue symbols show the fit for specialists, the red symbols show the fit for generalists. Black lines are 100 assemblages with 79 randomly selected generalists, used as a comparison to specialists when the same number of species of specialists and generalists are used.

Irrespective of the transformation or standardization, we always found a higher cross-validatory fit for the prediction of specialists than for generalists when we used the local environmental factors (Fig. 3). Nevertheless, the difference in the cross-validatory fit between generalists and specialists differed between transformations, with the lowest differences for the raw data and the log-transformed data. Furthermore, for these two transformations, the differences were due to the size of the matrices (see Fig. 3, cf. black lines and red symbols): more than 5% of the randomly reduced data sets of generalists showed a cross-validatory fit similar to that of the specialists.

**Figure 4:**
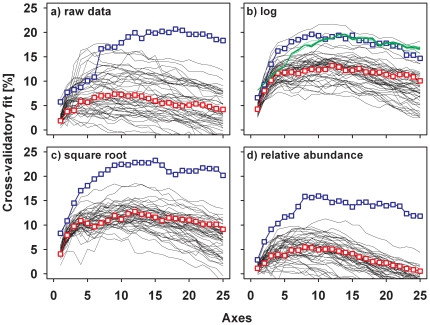
Cross-validatory fit of the raw data set of the composition of moth species and of three types of transformed data using the composition of the vegetation as the predictor. The fit is plotted against the number of ordination axes. The blue symbols show the fit for specialists, the red for generalists. Black lines are 100 assemblages with 79 randomly selected generalists, used as a comparison to specialists when the same number of species of specialists and generalists are used. The green line in (b) presents the mean cross-validatory fit of the correspondence analyses in which the host species of each moth was successively removed.

**Figure 5:**
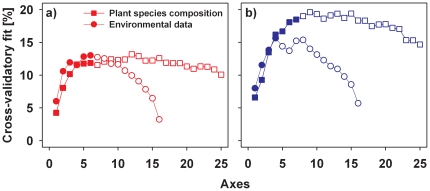
Cross-validatory fit of the log(x+1) transformed data of moth (a) generalists and (b) specialists plotted against the number of ordination axes for two sets of predictor variables: composition of the vegetation (squares) using co-correspondence analysis, and environmental variables (circles) using predictive canonical correspondence analysis. The filled symbols indicate significant axes according to permutation tests. For differences in the cross-validatory fit between data sets, we permuted residuals between data sets.

In contrast, for all transformations, the predictability of assemblages of specialists according to the composition of the vegetation was higher than the predictability of assemblages of generalists (Fig. 4). When we used the vegetation as the predictor matrix, the lowest difference in the predictability was found for log-transformed data; therefore, in all further analyses, we used the log-transformed data as a conservative approach. When we compared the two predictor sets for specialists and generalists, the composition of the vegetation always had a higher cross-validatory fit than the environmental data set (Fig. 5). However, for generalists, the difference in the maximum cross-validatory fits using plant species and environmental data was not significant (p = 0.9), whereas for specialists, the predictive power of plant species was significantly higher than the predictive power of the environmental data (p = 0.02). When we used log-transformed data, these differences between specialists and generalists was also consistent across the five periods of around five years (Fig. S4, Table S5).

The division of our moth assemblages according to generalists *versus* specialists is only one possibility. Other possible criteria for categorizing the assemblages include abundance, taxonomy and host life form (Table 1). For rare species with a restricted distribution (occupancy <29 reserves, the median of all occupancy values), we found a lower predictability than for common, more widespread species. However, the difference was low (Table 1). When we compared moth families (noctuids versus geometrids) as well as subsets generated according to the host life form, we again found only small differences in the predictability of the respective pairs of assemblages. But note that these comparisons are only suggestive as the predictability depends on the species within each matrix (see above).

**Table 1:** Cross-validatory fit of co-correspondence models for various subsets of our matrix of moth abundances (species occurring in at least 5 reserves) across 96 forest reserves in Bavaria (see Fig. 1).

	Matrices of		Difference in cross-validatory fit
Host specialisation	generalists	specialists	
	13.1 (492)	19.6 (79)	6.5
Taxonomy	noctuids	geometrids	
	13.8 (228)	15.1 (239)	1.4
Abundance	rare	common	
rare species occurring in <29 reserves	10.4 (290)	14.2 (281)	3.8
Host life form	tree	herb	
	13.4 (253)	14.4 (318)	1.0

In brackets we give the number of moth species in the matrix. Note that the largest difference in the cross-validatory fit appears for a subdivision of the original matrix into specialist and generalists.

**Figure 6:**
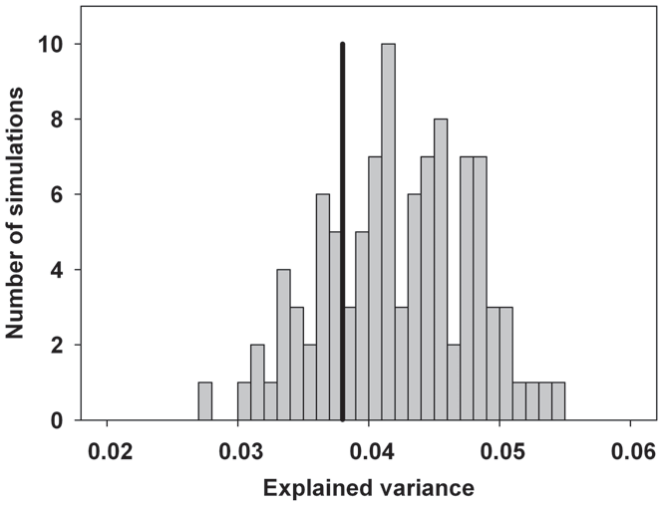
Mean explained variance (%) of single species co-correspondence analyses of 79 host-plant specialists (black line) compared to the mean explained variance expected from co-correspondence analyses with a randomly selected plant or plants as predictors. Each bar represents 100 runs.

The cross-validatory fit for specialists according to the composition of the vegetation, however, was not specifically due to the occurrence of host plants. We obtained similar mean cross-validatory fits of the predictive correspondence analyses when the host species of each moth was successively removed (green lines in Fig. 4b). Also, the mean explained variance of the single-species co-correspondence analysis of host-plant specialists was in the range expected for a randomly selected plant or plants as predictors (Fig. 6).

## Discussion

Our knowledge of insect assemblages living on a single plant species has made considerable progress through the use of compilations of faunal lists (older literature reviewed in [16]); see also [50], [51]. Despite these efforts, patterns of insect assemblages beyond a single plant species are still poorly understood [52], [53], [54]. Many studies have used species richness as a measure of α-diversity (e.g. [55]) and correlated species richness of phytophages with species richness of plants. The rationale behind such tests is that more plant species provide more hosts, and therefore more specialists can live in a habitat with many plant species, thereby increasing the overall species richness. The results of empirical tests, however, have been mixed [56], [57], [58]. Although species richness is a popular measure in community ecology, this variable ignores the species identity, host plant relationships as well as the potential of regional processes influencing local species richness (for example, the positive relationship between regional and local species richness [59].

Studies comparing species turnover between assemblages of insects have used either a measure of β-diversity or one of the many ordination techniques (e.g. [54], [60], [61]; Fig. S2). Again, β-diversity is an anonymous measure that ignores the species identity. Ordination techniques, in contrast, offer the possibility to consider species identity and abundance [39]. Until recently, nearly all of the studies on the diversity of phytophagous insects did not attempt to predict species composition within a habitat patch. In most of the published studies, canonical correspondence analysis or redundancy analyses were used to search for patterns in the compositions of assemblages (e.g. [60]). Such analyses do not really show whether the relationships have predictive power, especially if there are many independent variables. Using predictive co-correspondence analysis and canonical correspondence analysis, we were able to show that the quantitative composition of insect assemblages can be predicted by environmental factors as well as by the composition of plant communities. Furthermore, we found that the predictability differed between insect assemblages of host-plant specialists and generalists. Finally, we suggest that these results can be understood using the metacommunity concept. Cross-validation is thereby a powerful tool to estimate the predictive power and to compare the predictive power of various data sets (see Table 1). At a first glance, the cross-validatory fit of the models seems to be very low. However, as discussed by Schaffers et al. [35], the cross-validatory fit as used in our study is simply another yardstick, which may even become negative when the predictions generated by the model are less accurate than those that arise from using the mean species composition of assemblages across sites.

A problem of many studies analysing assemblages of plants and particularly animals along large spatial scales is sampling [62]. Time constraints do not always allow sampling in all years at all selected sites. This often leads to the situation that only few sites are available for statistical analysis [60]. In our case, we decided to sum the data across trapping nights, even though trapping varied considerably across sites and years. However, when we separated our data set into periods of around five years, the results appeared to be robust, although not always significant. This is in part due to low samples sizes within certain time periods. Overall, we are confident that our results are not influenced by the sampling of sites (Figs. S2 and S3). Furthermore, Table 1 shows that occurrence as well as abundance has some impact on the cross-validatory fit: as expected by common sense, the cross-validatory fit for rare species is lower than that for common species. Note that host-plant specialists are on average less abundant than host-plant generalists, but the cross-validatory fit for the specialists is even larger than for generalists. Therefore, the observed difference in the cross-validatory fit between generalists and specialists is even a conservative estimate.

In line with arguments concerning the evolution of habitat specialization and dispersal, we found that local environmental factors are more important for the predictability of specialists than for the predictability of generalists. Komonen et al. [26] found clear evidence that the mobility of butterflies with a narrow host range is lower than the mobility of butterflies with a wide host range. These authors argue that dispersal is risky for the specialists because of the problem of spotting habitats with suitable hosts, and therefore specialists should show a low dispersal rate. If the dispersal to and from a habitat is low, the abundance of species depends on local factors, which increases the predictability of the species according to the local habitat conditions as long as habitat conditions are fairly stable. This suggests that assemblages of host-plant specialists may resemble metacommunities, where species sort according to the habitat conditions [10]. Note that we analysed adult moths and therefore the dispersing life stage. However, we are not sure whether all individuals recorded during trapping reproduced in the sampled reserve. Some individuals from other areas may have been attracted by the general habitat conditions. If suitable host plants are lacking, these individuals are expected to leave the habitat patch.

Host plants have different life spans, and dynamics of host availability may differ considerably between species using herbs (short-lived hosts, high variability of host availability with patches) and those using trees (long-lived hosts, low variability of host availability with patches). If one considers that host plants are habitats [16] and habitats are the template for the evolution of dispersal [63], one might expect a higher propensity for dispersal in species using short-lived habitats compared to species using long-lived habitats [64]. Therefore, following our line of arguments within the introduction, we would expect a higher predictability for moth species using long-lived hosts than for those using short-lived hosts. However, we did not find this pattern, which suggests that host specialization is more important than host type for predicting local assemblages of moths. Nevertheless, the reasons for this result are far from clear and may suggest that the observed difference between host-plant generalists and specialists is not only based on dispersal that evolved in response to the spatial predictability of host plants.

Furthermore, it is not self-evident whether the environmental variables we used in our analysis are really relevant for the distribution and abundance of insect species (see also [65]). In analyses of insect communities along road verges, Schaffers et al. [35] found that the composition of plant communities was a much better predictor of insect and spider assemblages than environmental variables or variables characterizing vegetation structure. In contrast to phytophages, spiders are not directly dependent on plant species, which suggests that the composition of the vegetation is a powerful surrogate for complex local habitat conditions that are not captured by the available environmental measurements [35]. In line with these results, we found that the difference between the predictability of host-plant specialists and generalists according to the local composition of the vegetation was consistent across transformations.

This difference in the predictability of host-plant specialists and generalists according to the composition of the vegetation may have a trivial explanation. In contrast to host-plant generalists, host-plant specialists need a particular host plant or a few host plants [19], [37]. This would explain the outperformance of the predictability of assemblages of host-plant specialist according to the composition of the vegetation compared to their predictability according to environmental factors (our second hypothesis stated in the Introduction). However, our more-detailed analyses suggested that this difference in the predictability of host-plant specialists according to environmental factors and the composition of the vegetation is not due to host-plant relationships, which contradicts our third hypothesis. Assemblages of host-plant specialists reacted differently than generalists to the environmental factors mirrored by the vegetation in that host-plant specialists seemed to be also habitat specialists. The species composition of the vegetation is thereby only a detailed mirror of the variation of environmental conditions within and between sites. Finally, the above arguments lead to the speculation that dispersing individuals first check the general habitat conditions and then check for the occurrence of the host plant. This is in line with studies on pierid butterflies, in which host-plant affiliations are strongly influenced by habitat characteristics [66]. Furthermore, a lower propensity of dispersal in phytophages with a narrow host range might lead to a lower level of gene flow compared to generalists [67]. Gene flow not only is important for the maintenance of genetic diversity [68], [69], but also disturbs the evolution of local adaptations. Therefore, host-plant specialists may be able to evolve adaptations to the habitat ([66]; for a general discussion of ecological specialization see [70]).

Our result that the predictability of assemblages differs between host-plant generalists and specialists is also in line with findings from species distribution models [71]. Several syntheses of such models have shown that the distributions of specialists are easier to predict than the distribution of generalists (e.g. [72], [73]). Most of these species distribution models considered only presence/absence data, whereas our approach predicts the quantitative composition of the assemblages. Furthermore, most species distribution models use environmental data, and species are divided into specialists and generalists according to the environment, which introduces some circularity. In contrast, we defined specialists and generalists according to independent host-plant information, and we predicted the occurrence and relative abundance of species.

During the long history of vegetation studies, much evidence has accumulated that the species composition of the vegetation mirrors complex habitat conditions [48]. We and others [35] found that the species composition of the vegetation is a good predictor of the composition of animal communities. Overall this supports the widespread use of the vegetation to map specific habitats and to establish networks of protected areas [74]. For example, within the framework of Natura 2000 in Europe, areas are selected according to floristic criteria and classifications of the vegetation [75]. In addition to the advantages of sampling the vegetation, the integrative nature of plant assemblages forms a general umbrella for conservation planning, although specialized groups of organisms, e.g. species living in dead wood, need special attention [76].

The metacommunity concept is a conceptual tool for understanding the theoretical underpinning of species assemblages to design powerful experiments (e.g. [12], [77]). This concept also forms a solid basis to understand the statistical patterns of insect assemblages across space or environmental gradients [78]. The test we provided differs from the framework suggested by [33] in that we infer the metacommunity structure not from certain characteristics of the community matrix (e.g. coherence, turnover, boundary clumping), but by using independent data on local environmental factors to predict assemblages. Yet caution is warranted: Firstly, the different metacommunity paradigms are only simplified conceptual models, and real assemblages sort in between these extremes. Nevertheless, the metacommunity concept expands earlier efforts to understand local assemblages by setting these assemblages into a regional context. Secondly, we only inferred the importance of regional processes (dispersal) from differences in local patterns of species assemblages. Therefore independent information on the propensity of dispersal or dispersal strategies of generalists and specialists is sorely needed to scrutinize our conclusions from a set of observational data (for an example with spiders see [79]).

## Supporting Information

Figure S1 (a) Scatter plot of species versus number of individuals collected for each of the 114 reserves. The line indicates the minimum of 500 individuals, which was selected for including a reserve. (b) Scatter plot of occupancy (number of reserves in which a species was recorded) *versus* number of sampled individuals of 820 moth species sampled in in our final selected 96 strict forest reserves in Bavaria. The line indicates the species occurring in less than 5 sites. Note the log transformation of the x-axis. (c) The number of species observed versus the number of species expected in 114 reserves. The latter was calculated as the mean of the two variants Chao (unbiased variant) and ACE of extrapolated richness in *estimateR* in the package vegan. The vertical line indicates the cutpoint of 500 individuals, which was selected for including a reserve in the final analysis. (d) The ratio of observed/expected species versus the observed species of 114 reserves. (e) Species versus number of trapping nights; reserves marked by black dots were removed in the final analysis. (f) Observed/expected species versus trapping nights; reserves marked by black dots were again removed in the final analysis (e).
(TIF)

Figure S2 **Percentage of moth species richness (Richness) and community diversity (Simpson, Shannon) explained by the alpha and beta components of diversity on four spatial and temporal scales: reserves within one period of ≈5 years (periods: 1980–1989, 1985–1889, 1990–1994, 1995–1999, 2000–2006), between periods, between reserves, and between ecoregions (** **Fig. 1** **).** The components were determined by additive partitioning of diversity using the function *adipart* within the package vegan (www.R-project.org). For each diversity measure, we calculated the components for generalists and specialists separately. Note that the beta-diversity components were always larger for specialists than for generalists. Further note that the diversity component between periods was generally low. Only species occurring in at least 5 reserves (the same as used in the main analysis) were included in the partitioning.
(TIF)

Figure S3 **Scatter plot of plant species versus number of relevés and histogram of plant relevés in the final 96 reserves.** Note that lowest number of plant species occur in sites with several relevés. Such sites are acidic beech plant communities, naturally poor in plant species.
(TIF)

Figure S4 **Cross-validatory fits for the prediction of assemblages of moth generalists (red) and specialists [blue; data log(x+1)-transformed] plotted against the number of ordination axes used for prediction of assemblages for periods of ≈5 years using only reserves that were sample for at least 2 nights per period.** We used two sets of predictor variables: composition of the vegetation using co-correspondence analysis, and environmental variables using predictive canonical correspondence analysis as in Fig. 6. Note that the results presented within the manuscript for the pooled data remain the same for all time periods (see also Table S2), although the number of reserves sampled within a period decreased (maximal decrease down to 14 reserves).
(TIF)

Table S1 **Distribution of nights of light trapping in the 96 forest reserves that entered the analysis.**
(DOC)

Table S2 **Data of 571 moths used in the analyses.**
(XLS)

Table S3 **Environmental raw data used for calculating the climate PCA scores and altitude, as well as coordinates.**
(XLS)

Table S4 **Presence/absence data of plants occurring in at least 5 reserves used for calculating the Ellenberg indicator values and as predictor data set.**
(XLS)

Table S5 **Maximum cross-validatory fit of the log(x+1) transformed matrix of moth assemblages for host-plant generalists and specialists using two sets of predictor variables (environmental variables and plant species composition) for periods of ≈5 years (see Fig. S4).** The *P*-value presents a test of the difference in the predictability of the various assemblages by the two data sets (see Material and Methods).
(DOC)
