# Supplementary figures and images for: The Predictability of Phytophagous Insect Communities: Host Specialists as Habitat Specialists
Source: PLoS One. 2011 Oct 7;6(10):e25986. doi: 10.1371/journal.pone.0025986 (PMC3189246; doi:10.1371/journal.pone.0025986)

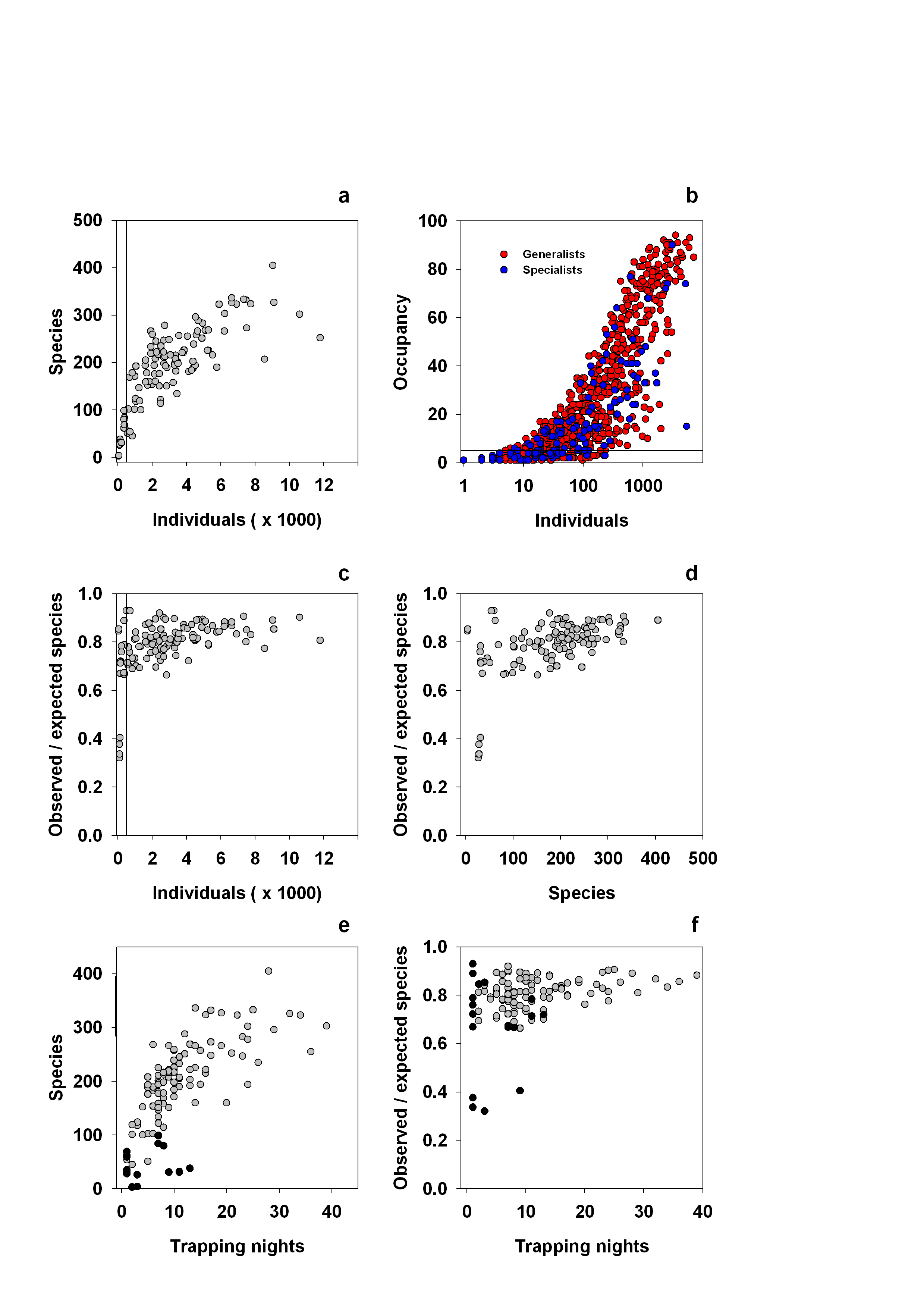

Supplement: Figure S1 — (a) Scatter plot of species versus number of individuals collected for each of the 114 reserves. The line indicates the minimum of 500 individuals, which was selected for including a reserve. (b) Scatter plot of occupancy (number of reserves in which a species was recorded) versus number of sampled individuals of 820 moth species sampled in in our final selected 96 strict forest reserves in Bavaria. The line indicates the species occurring in less than 5 sites. Note the log transformation of the x-axis. (c) The number of species observed versus the number of species expected in 114 reserves. The latter was calculated as the mean of the two variants Chao (unbiased variant) and ACE of extrapolated richness in estimateR in the package vegan. The vertical line indicates the cutpoint of 500 individuals, which was selected for including a reserve in the final analysis. (d) The ratio of observed/expected species versus the observed species of 114 reserves. (e) Species versus number of trapping nights; reserves marked by black dots were removed in the final analysis. (f) Observed/expected species versus trapping nights; reserves marked by black dots were again removed in the final analysis (e). (TIF) [file pone.0025986.s001.tif]

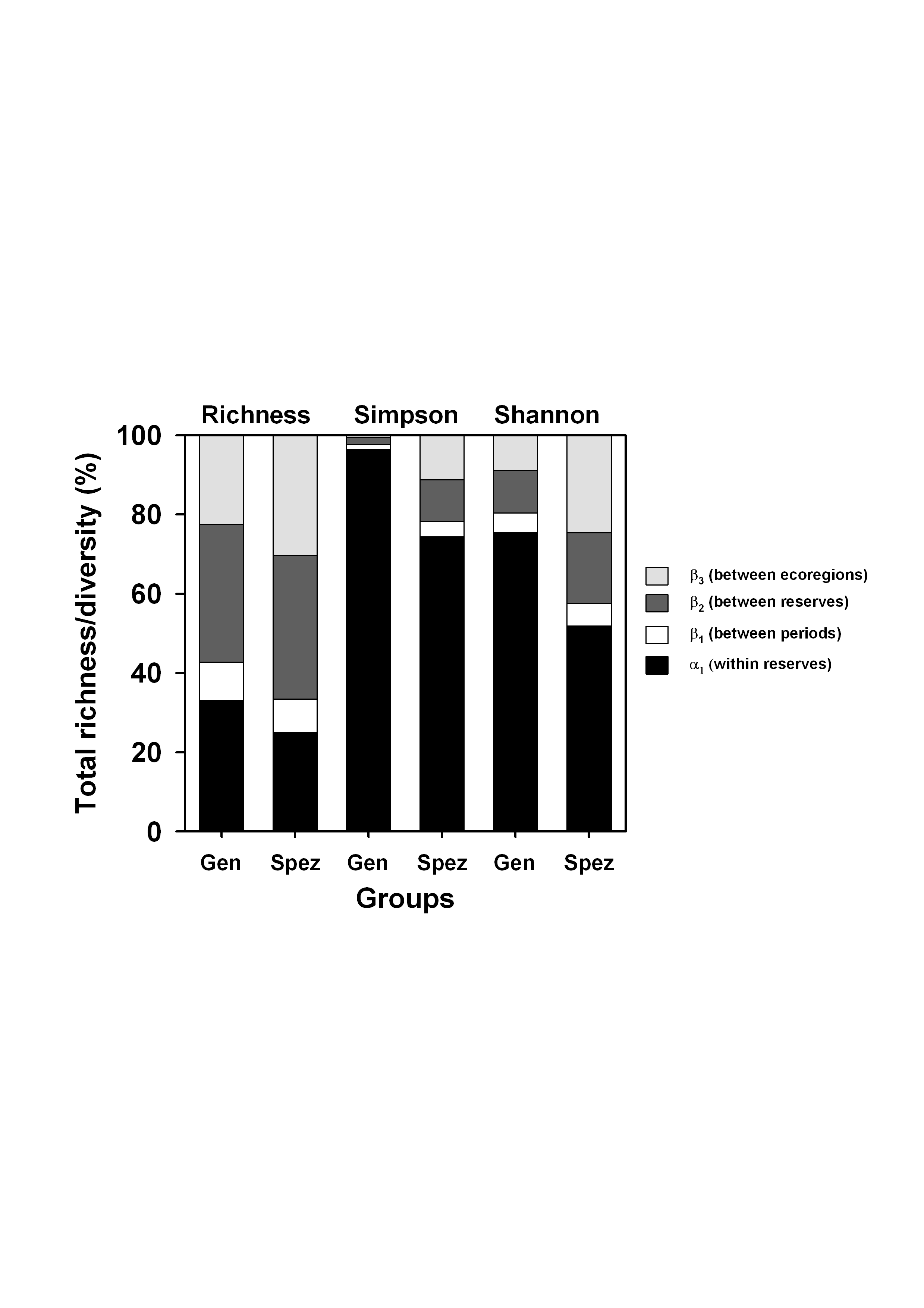

Supplement: Figure S2 — Percentage of moth species richness (Richness) and community diversity (Simpson, Shannon) explained by the alpha and beta components of diversity on four spatial and temporal scales: reserves within one period of ≈5 years (periods: 1980–1989, 1985–1889, 1990–1994, 1995–1999, 2000–2006), between periods, between reserves, and between ecoregions ( Fig. 1 ). The components were determined by additive partitioning of diversity using the function adipart within the package vegan (www.R-project.org). For each diversity measure, we calculated the components for generalists and specialists separately. Note that the beta-diversity components were always larger for specialists than for generalists. Further note that the diversity component between periods was generally low. Only species occurring in at least 5 reserves (the same as used in the main analysis) were included in the partitioning. (TIF) [file pone.0025986.s002.tif]

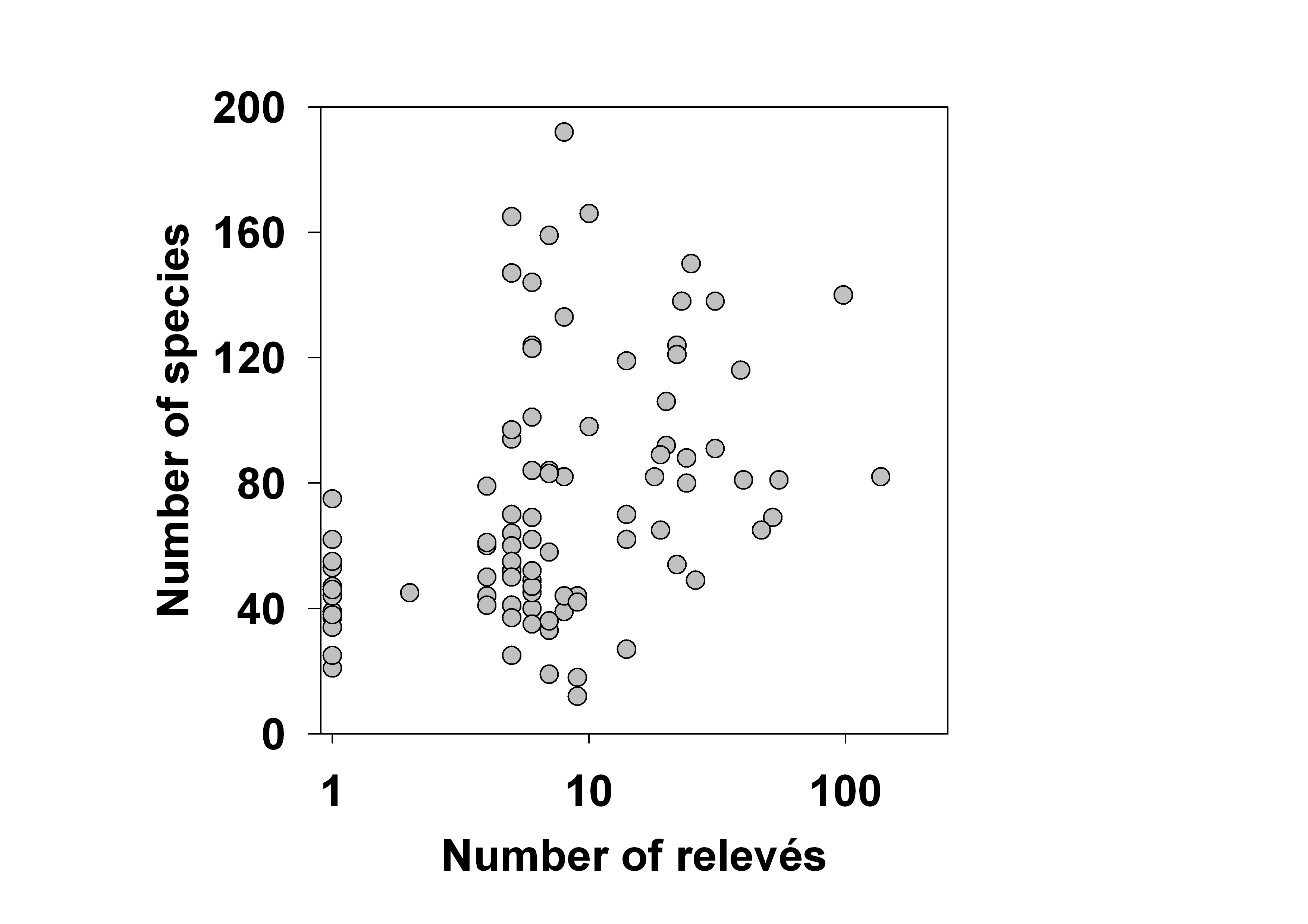

Supplement: Figure S3 — Scatter plot of plant species versus number of relevés and histogram of plant relevés in the final 96 reserves. Note that lowest number of plant species occur in sites with several relevés. Such sites are acidic beech plant communities, naturally poor in plant species. (TIF) [file pone.0025986.s003.tif]

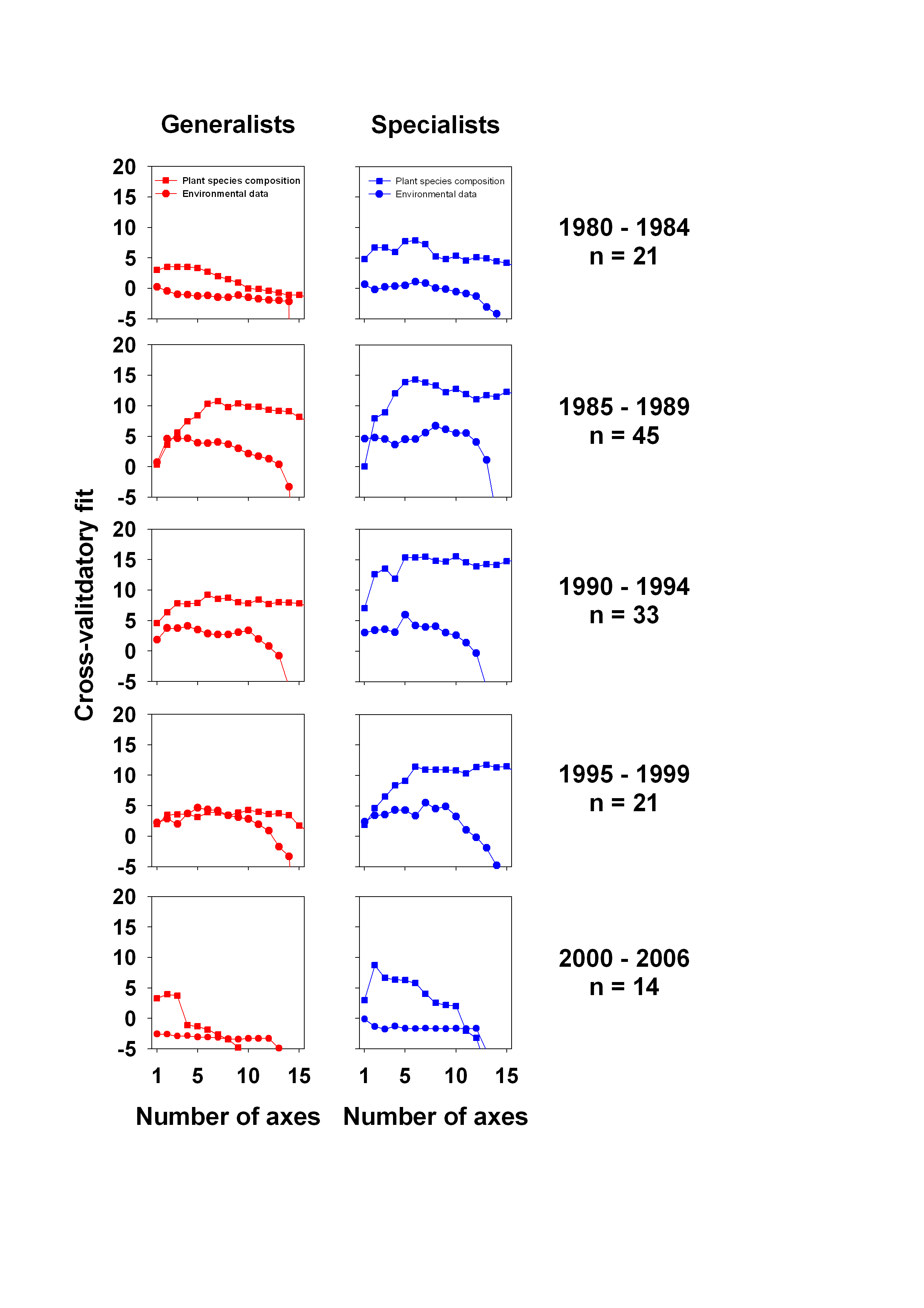

Supplement: Figure S4 — Cross-validatory fits for the prediction of assemblages of moth generalists (red) and specialists [blue; data log(x+1)-transformed] plotted against the number of ordination axes used for prediction of assemblages for periods of ≈5 years using only reserves that were sample for at least 2 nights per period. We used two sets of predictor variables: composition of the vegetation using co-correspondence analysis, and environmental variables using predictive canonical correspondence analysis as in Fig. 6. Note that the results presented within the manuscript for the pooled data remain the same for all time periods (see also Table S2), although the number of reserves sampled within a period decreased (maximal decrease down to 14 reserves). (TIF) [file pone.0025986.s004.tif]
