# Supplementary material for: The Predictability of Phytophagous Insect Communities: Host Specialists as Habitat Specialists
Source: PLoS One. 2011 Oct 7;6(10):e25986. doi: 10.1371/journal.pone.0025986 (PMC3189246; doi:10.1371/journal.pone.0025986)
Supplement: Table S1 — Distribution of nights of light trapping in the 96 forest reserves that entered the analysis. (DOC) [file pone.0025986.s005.doc]

**Table S1:** Distribution of nights of light trapping in the 96 forest reserves that entered the analysis.

| Reserve number | Sum of nights | 1980 | 1982 | 1983 | 1984 | 1985 | 1986 | 1987 | 1988 | 1989 | 1990 | 1991 | 1992 | 1993 | 1994 | 1995 | 1996 | 1997 | 1998 | 1999 | 2000 | 2001 | 2002 | 2003 | 2004 | 2005 | 2006 |
| --- | --- | --- | --- | --- | --- | --- | --- | --- | --- | --- | --- | --- | --- | --- | --- | --- | --- | --- | --- | --- | --- | --- | --- | --- | --- | --- | --- |
| 1 | 10 |  |  |  |  |  |  | 1 | 1 | 2 | 1 | 3 | 1 |  | 1 |  |  |  |  |  |  |  |  |  |  |  |  |
| 3 | 12 |  |  |  |  |  | 2 | 2 | 3 |  |  | 4 |  | 1 |  |  |  |  |  |  |  |  |  |  |  |  |  |
| 4 | 14 |  |  |  |  |  | 1 | 4 | 4 |  |  | 4 |  | 1 |  |  |  |  |  |  |  |  |  |  |  |  |  |
| 5 | 6 |  |  |  |  |  |  | 1 | 1 | 1 |  | 1 | 1 | 1 |  |  |  |  |  |  |  |  |  |  |  |  |  |
| 6 | 10 |  |  |  |  |  |  | 1 | 2 | 2 | 1 | 3 | 1 |  |  |  |  |  |  |  |  |  |  |  |  |  |  |
| 7 | 17 |  |  |  |  |  | 3 | 1 | 2 | 3 | 1 | 3 | 1 | 3 |  |  |  |  |  |  |  |  |  |  |  |  |  |
| 8 | 9 |  |  |  |  |  |  | 3 | 2 | 1 |  |  |  | 3 |  |  |  |  |  |  |  |  |  |  |  |  |  |
| 10 | 8 |  |  |  |  |  |  | 4 | 2 | 1 |  |  |  | 1 |  |  |  |  |  |  |  |  |  |  |  |  |  |
| 11 | 7 |  |  |  |  |  |  | 2 | 1 | 1 |  | 1 | 1 | 1 |  |  |  |  |  |  |  |  |  |  |  |  |  |
| 12 | 25 |  |  |  |  |  | 3 | 7 | 4 | 3 | 1 |  | 3 |  |  |  |  |  |  | 4 |  |  |  |  |  |  |  |
| 13 | 17 |  |  |  |  |  | 4 | 6 | 4 | 3 |  |  |  |  |  |  |  |  |  |  |  |  |  |  |  |  |  |
| 15 | 12 |  |  |  |  |  |  | 3 |  | 5 | 2 | 1 |  |  | 1 |  |  |  |  |  |  |  |  |  |  |  |  |
| 16 | 24 |  |  |  |  |  |  | 9 |  | 8 | 4 | 1 |  |  |  | 1 |  |  |  |  |  |  |  | 1 |  |  |  |
| 17 | 6 |  |  |  |  |  |  |  |  |  |  |  |  |  |  |  |  |  |  | 5 |  |  |  | 1 |  |  |  |
| 21 | 7 |  |  |  |  |  |  |  |  |  |  |  | 1 |  |  |  |  | 2 |  | 4 |  |  |  |  |  |  |  |
| 22 | 10 |  |  |  |  |  |  |  |  |  |  |  | 2 |  |  |  |  | 3 |  | 5 |  |  |  |  |  |  |  |
| 25 | 7 |  |  |  |  |  |  |  |  |  |  |  |  |  |  | 3 |  | 1 |  | 2 |  |  |  | 1 |  |  |  |
| 26 | 8 |  |  |  |  |  |  |  |  |  |  |  |  | 1 |  | 1 |  | 2 |  | 4 |  |  |  |  |  |  |  |
| 32 | 36 |  |  |  |  |  |  |  | 13 | 12 |  | 11 |  |  |  |  |  |  |  |  |  |  |  |  |  |  |  |
| 33 | 4 |  |  |  |  |  |  |  |  |  |  |  | 1 |  |  |  |  | 2 |  | 1 |  |  |  |  |  |  |  |
| 35 | 2 |  |  |  |  |  |  |  |  |  |  |  |  |  |  |  |  |  |  | 2 |  |  |  |  |  |  |  |
| 36 | 3 |  |  |  |  |  |  |  |  |  |  |  |  |  |  |  |  |  |  | 3 |  |  |  |  |  |  |  |
| 38 | 38 |  |  |  |  |  |  |  |  | 3 | 3 | 1 |  | 1 |  | 13 | 15 |  |  |  |  |  | 1 | 1 |  |  |  |
| 39 | 10 |  |  |  |  |  |  |  |  | 2 | 3 | 1 |  | 2 |  | 2 |  |  |  |  |  |  |  |  |  |  |  |
| 40 | 13 |  |  | 5 | 5 |  |  |  |  |  |  |  |  |  |  |  |  |  |  |  |  |  |  |  |  |  | 3 |
| 41 | 11 |  |  | 3 | 8 |  |  |  |  |  |  |  |  |  |  |  |  |  |  |  |  |  |  |  |  |  |  |
| 43 | 10 |  | 2 | 5 | 2 |  |  |  |  |  |  |  |  |  | 1 |  |  |  |  |  |  |  |  |  |  |  |  |
| 44 | 34 |  | 1 | 5 | 2 |  |  |  |  |  |  |  |  | 6 | 12 | 8 |  |  |  |  |  |  |  |  |  |  |  |
| 45 | 9 |  |  | 4 | 5 |  |  |  |  |  |  |  |  |  |  |  |  |  |  |  |  |  |  |  |  |  |  |
| 46 | 24 |  | 3 | 4 | 6 |  |  |  |  |  |  |  |  |  |  |  | 11 |  |  |  |  |  |  |  |  |  |  |
| 47 | 10 |  |  | 2 | 8 |  |  |  |  |  |  |  |  |  |  |  |  |  |  |  |  |  |  |  |  |  |  |
| 48 | 15 |  | 1 | 5 | 2 |  |  | 7 |  |  |  |  |  |  |  |  |  |  |  |  |  |  |  |  |  |  |  |
| 49 | 32 |  |  | 4 | 6 |  |  |  |  |  |  |  |  |  |  | 2 | 1 |  |  |  |  |  | 11 | 5 | 3 |  |  |
| 50 | 8 | 1 |  | 1 | 6 |  |  |  |  |  |  |  |  |  |  |  |  |  |  |  |  |  |  |  |  |  |  |
| 51 | 11 |  | 5 | 4 | 2 |  |  |  |  |  |  |  |  |  |  |  |  |  |  |  |  |  |  |  |  |  |  |
| 52 | 5 |  |  | 2 | 3 |  |  |  |  |  |  |  |  |  |  |  |  |  |  |  |  |  |  |  |  |  |  |
| 53 | 6 |  |  | 3 | 3 |  |  |  |  |  |  |  |  |  |  |  |  |  |  |  |  |  |  |  |  |  |  |
| 55 | 4 |  |  |  |  |  |  | 1 |  |  |  |  |  |  |  |  | 2 | 1 |  |  |  |  |  |  |  |  |  |
| 56 | 18 |  |  |  |  |  |  | 3 |  |  |  |  | 1 |  |  |  | 3 |  |  |  | 3 | 4 | 2 | 2 |  |  |  |
| 57 | 14 |  |  |  |  |  |  | 3 | 1 | 1 |  | 1 |  |  |  |  |  | 5 |  | 3 |  |  |  |  |  |  |  |
| 59 | 2 |  |  |  |  |  |  |  |  | 1 |  |  |  |  |  |  | 1 |  |  |  |  |  |  |  |  |  |  |
| 61 | 16 |  |  |  |  |  |  |  |  |  |  |  |  |  |  | 1 |  |  | 8 | 2 | 4 |  |  | 1 |  |  |  |
| 63 | 11 |  |  |  |  |  |  |  |  | 5 | 1 | 2 | 1 |  |  |  |  |  |  |  |  |  |  |  |  | 1 | 1 |
| 65 | 8 |  |  |  |  |  |  |  |  | 2 | 2 | 2 | 1 |  |  |  |  |  |  |  |  |  |  |  |  |  | 1 |
| 66 | 7 |  |  |  |  |  |  |  |  |  |  |  |  | 2 |  | 2 |  |  |  | 3 |  |  |  |  |  |  |  |
| 67 | 7 |  |  |  |  |  |  |  |  |  |  |  | 1 | 2 |  | 2 |  |  |  | 2 |  |  |  |  |  |  |  |
| 69 | 26 |  |  |  |  |  |  |  |  |  |  |  |  | 2 |  |  |  |  |  |  |  |  |  | 21 | 3 |  |  |
| 71 | 5 |  |  |  |  |  |  |  |  |  |  |  |  |  |  |  |  |  |  |  |  |  |  | 1 | 4 |  |  |
| 73 | 12 |  |  |  |  |  |  |  |  | 5 | 3 | 2 | 1 |  |  |  |  |  |  |  |  |  |  |  |  |  | 1 |
| 75 | 23 |  |  |  |  |  |  |  | 17 | 6 |  |  |  |  |  |  |  |  |  |  |  |  |  |  |  |  |  |
| 76 | 5 |  |  |  |  |  |  |  |  |  |  |  |  |  | 1 | 1 | 1 |  |  |  |  |  |  | 2 |  |  |  |
| 77 | 16 |  |  |  |  |  |  | 4 | 4 | 3 |  | 2 |  |  |  | 3 |  |  |  |  |  |  |  |  |  |  |  |
| 78 | 9 |  |  |  |  |  |  |  |  |  |  |  |  |  |  |  |  | 3 |  | 6 |  |  |  |  |  |  |  |
| 81 | 4 |  |  |  |  |  |  |  |  |  |  |  |  |  |  |  |  | 4 |  |  |  |  |  |  |  |  |  |
| 84 | 3 |  |  |  |  |  |  |  |  |  |  |  |  |  |  |  |  | 3 |  |  |  |  |  |  |  |  |  |
| 85 | 6 |  |  |  |  |  |  |  |  | 1 | 1 |  |  |  |  |  |  | 4 |  |  |  |  |  |  |  |  |  |
| 86 | 5 |  |  |  |  |  |  |  |  | 1 | 1 |  |  |  |  |  |  | 3 |  |  |  |  |  |  |  |  |  |
| 87 | 5 |  |  |  |  |  | 2 | 3 |  |  |  |  |  |  |  |  |  |  |  |  |  |  |  |  |  |  |  |
| 89 | 7 |  |  |  |  |  | 2 |  |  |  |  | 1 | 1 |  |  | 3 |  |  |  |  |  |  |  |  |  |  |  |
| 90 | 9 |  |  |  |  |  |  |  |  |  |  |  |  | 5 |  | 3 | 1 |  |  |  |  |  |  |  |  |  |  |
| 91 | 7 |  |  |  |  |  |  |  |  |  |  |  |  | 3 |  | 2 | 2 |  |  |  |  |  |  |  |  |  |  |
| 94 | 7 |  |  |  |  |  |  |  |  |  |  |  | 1 |  | 1 | 3 | 2 |  |  |  |  |  |  |  |  |  |  |
| 95 | 11 |  |  |  |  |  |  |  |  |  |  |  | 2 |  | 2 | 5 | 1 |  |  |  |  |  |  |  |  |  | 1 |
| 100 | 19 |  |  |  |  |  |  | 1 |  | 4 |  |  | 1 |  |  |  | 3 | 4 |  | 5 |  |  |  |  |  |  | 1 |
| 101 | 13 |  |  |  |  |  |  | 2 |  | 2 |  |  |  |  |  |  | 3 | 4 |  | 2 |  |  |  |  |  |  |  |
| 104 | 14 |  |  |  |  |  |  |  |  |  |  |  | 2 |  | 1 | 6 | 4 | 1 |  |  |  |  |  |  |  |  |  |
| 105 | 10 |  |  |  |  |  |  |  |  |  |  |  |  |  |  | 2 |  | 6 | 1 | 1 |  |  |  |  |  |  |  |
| 106 | 10 |  |  |  |  |  |  |  |  |  |  |  |  | 1 | 1 |  |  | 6 |  | 2 |  |  |  |  |  |  |  |
| 107 | 11 |  |  |  |  |  |  |  |  |  |  |  | 1 |  | 2 | 5 | 2 |  |  | 1 |  |  |  |  |  |  |  |
| 111 | 9 |  |  |  |  |  |  |  |  |  |  |  |  |  | 3 |  |  | 4 |  | 2 |  |  |  |  |  |  |  |
| 116 | 7 |  |  |  | 2 |  | 5 |  |  |  |  |  |  |  |  |  |  |  |  |  |  |  |  |  |  |  |  |
| 117 | 7 |  |  |  |  |  | 7 |  |  |  |  |  |  |  |  |  |  |  |  |  |  |  |  |  |  |  |  |
| 118 | 29 |  |  |  | 10 | 4 | 12 | 3 |  |  |  |  |  |  |  |  |  |  |  |  |  |  |  |  |  |  |  |
| 119 | 10 |  |  | 6 | 4 |  |  |  |  |  |  |  |  |  |  |  |  |  |  |  |  |  |  |  |  |  |  |
| 120 | 11 |  |  | 3 | 2 |  | 6 |  |  |  |  |  |  |  |  |  |  |  |  |  |  |  |  |  |  |  |  |
| 121 | 9 |  |  | 2 | 1 |  | 6 |  |  |  |  |  |  |  |  |  |  |  |  |  |  |  |  |  |  |  |  |
| 122 | 8 |  |  |  | 1 | 1 | 6 |  |  |  |  |  |  |  |  |  |  |  |  |  |  |  |  |  |  |  |  |
| 123 | 17 |  |  |  | 5 | 4 | 8 |  |  |  |  |  |  |  |  |  |  |  |  |  |  |  |  |  |  |  |  |
| 124 | 16 |  |  |  | 3 | 7 | 6 |  |  |  |  |  |  |  |  |  |  |  |  |  |  |  |  |  |  |  |  |
| 125 | 7 |  |  |  |  |  | 7 |  |  |  |  |  |  |  |  |  |  |  |  |  |  |  |  |  |  |  |  |
| 126 | 13 |  |  |  | 1 | 4 | 8 |  |  |  |  |  |  |  |  |  |  |  |  |  |  |  |  |  |  |  |  |
| 129 | 8 |  |  |  |  |  | 6 |  |  | 2 |  |  |  |  |  |  |  |  |  |  |  |  |  |  |  |  |  |
| 130 | 8 |  |  |  |  |  | 6 |  |  | 2 |  |  |  |  |  |  |  |  |  |  |  |  |  |  |  |  |  |
| 131 | 28 |  |  |  | 2 | 3 | 15 | 2 |  |  |  |  | 1 | 2 |  |  |  |  |  | 2 |  |  |  |  |  |  | 1 |
| 132 | 22 |  |  |  | 1 |  | 19 |  |  |  |  |  |  |  |  |  |  |  |  | 1 |  |  |  |  |  |  | 1 |
| 133 | 19 |  |  |  |  |  | 9 |  |  |  |  |  |  |  |  |  |  |  |  | 2 |  |  |  | 8 |  |  |  |
| 134 | 15 |  |  |  | 1 | 2 | 12 |  |  |  |  |  |  |  |  |  |  |  |  |  |  |  |  |  |  |  |  |
| 135 | 14 |  |  |  | 1 |  | 12 |  |  |  |  |  |  |  |  |  |  |  |  | 1 |  |  |  |  |  |  |  |
| 138 | 7 |  |  |  |  |  |  |  |  |  |  |  |  | 3 | 1 | 2 |  |  |  | 1 |  |  |  |  |  |  |  |
| 144 | 7 |  |  |  |  |  |  |  |  |  |  |  |  |  | 1 |  | 1 | 4 |  | 1 |  |  |  |  |  |  |  |
| 146 | 1 |  |  |  |  |  |  |  |  |  |  |  |  |  | 1 |  |  |  |  |  |  |  |  |  |  |  |  |
| 148 | 20 |  |  |  |  |  |  |  |  |  |  |  |  |  |  | 1 | 2 | 8 | 3 | 6 |  |  |  |  |  |  |  |
| 154 | 1 |  |  |  |  |  |  |  |  |  |  |  |  |  |  |  |  |  |  |  |  |  |  | 1 |  |  |  |
| 155 | 8 |  |  |  |  |  |  |  |  |  |  |  |  |  |  |  |  |  |  |  |  |  |  | 2 | 6 |  |  |
| 160 | 5 |  |  |  |  |  |  |  |  |  |  |  |  |  |  |  |  |  |  | 3 |  |  |  | 2 |  |  |  |
| 161 | 6 |  |  |  |  |  |  |  |  |  |  |  |  |  |  |  |  |  |  |  |  |  |  | 6 |  |  |  |
