# Supplementary material for: The Predictability of Phytophagous Insect Communities: Host Specialists as Habitat Specialists
Source: PLoS One. 2011 Oct 7;6(10):e25986. doi: 10.1371/journal.pone.0025986 (PMC3189246; doi:10.1371/journal.pone.0025986)
Supplement: Table S5 — Maximum cross-validatory fit of the log(x+1) transformed matrix of moth assemblages for host-plant generalists and specialists using two sets of predictor variables (environmental variables and plant species composition) for periods of ≈5 years (see Fig. S4). The P-value presents a test of the difference in the predictability of the various assemblages by the two data sets (see Material and Methods). (DOC) [file pone.0025986.s009.doc]

**Table S5:** Maximum cross-validatory fit of the log(x+1) transformed matrix of moth assemblages for host-plant generalists and specialists using two sets of predictor variables (environmental variables and plant species composition) for periods of ≈ 5 years (see Fig. S4). The *P*-value presents a test of the difference in the predictability of the various assemblages by the two data sets (see Material and Methods).

| Period | Generalists | | | Specialists | | |
| --- | --- | --- | --- | --- | --- | --- |
|  | Environment | Plants | *P* | Environment | Plants | *P* |
| 1980–1984 | 0.23 | 3.5 | 0.001 | 1.1 | 7.82 | 0.002 |
| 1985–1989 | 4.6 | 10.7 | 0.015 | 6.7 | 14.3 | 0.001 |
| 1990–1994 | 4.1 | 9.2 | 0.001 | 6.0 | 16.9 | 0.030 |
| 1995–1999 | 4.7 | 4.2 | 0.922 | 5.5 | 11.7 | 0.040 |
| 2000–2006 | −2.6 | 3.9 | 0.060 | −0.1 | 8.7 | 0.071 |
